# Fit for Purpose—Re-Designing Australia’s Mental Health Information System

**DOI:** 10.3390/ijerph19084808

**Published:** 2022-04-15

**Authors:** Sebastian Rosenberg, Luis Salvador-Carulla, Graham Meadows, Ian Hickie

**Affiliations:** 1 Brain and Mind Centre, University of Sydney, Level 4, 94 Mallett Street, Sydney, NSW 2050, Australia; ian.hickie@sydney.edu.au; 2 Mental Health Policy Unit, Health Research Institute, Faculty of Health, University of Canberra, Canberra, ACT 2617, Australia; luis.salvador-carulla@canberra.edu.au; 3 Department of Psychiatry, Monash Health, Melbourne, VIC 3168, Australia; graham.meadows@monash.edu

**Keywords:** mental health, accountability, quality improvement, policy development

## Abstract

Background: Monitoring and reporting mental health is complex. Australia’s first National Mental Health Strategy in 1992 included a new national commitment to accountability and data collection in mental health. This article provides a narrative review of thirty years of experience. Materials and Methods: This review considers key documents, policies, plans and strategies in relation to the evolution of mental health data and reporting. Documents produced by the Federal and the eight state and territory governments are considered, as well as publications produced by key information agencies, statutory authorities and others. A review of this literature demonstrates both its abundance and limitations. Results: Australia’s approach to mental health reporting is characterised by duplication and a lack of clarity. The data available fail to do justice to the mental health services provided in Australia. Mental health data collection and reporting processes are centrally driven, top–down and activity-focused, largely eschewing actual health outcomes, the social determinants of mental health. There is little, if any, link to clearly identifiable service user or carer priorities. Consequently, it is difficult to link this process longitudinally to clinical or systemic quality improvement. Initial links between the focus of national reform efforts and mental health data collection were evident, but these links have weakened over time. Changes to governance and reporting, including under COVID, have made the task of delivering accountability for mental health more difficult. Conclusion: Australia’s current approach is not fit for purpose. It is at a pivotal point in mental health reform, with new capacity to use modelled data to simulate prospective mental health reform options. By drawing on these new techniques and learning the lessons of the past, Australia (and other nations) can design and implement more effective systems of planning, reporting and accountability for mental health.

## 1. Introduction

What does effective national monitoring and reporting of mental health care look like? The year 2022 is the thirtieth anniversary of Australia’s National Mental Health Strategy, which implemented a new process for data collection as a central function to drive better accountability for mental health services [1].

This narrative review attempts to assess the extent to which Australia’s efforts have yielded an effective system of accountability for mental health. This assessment is problematic. There has never been any formal evaluation of the strategy overall. Initial markers of success were not described to permit simple evaluation of progress. Evidence of impact, if available at all, is typically qualitative or summative, not quantitative.

There are some strengths, but also many weaknesses, in the approach taken. This has delivered an Australian reporting system which predominantly focuses on administrative data, inputs and outputs. Much is known about budgets, the number of occupied beds and outpatient occasions of services. We know staffing numbers and costs. However, few details are known about who is presenting for mental health care and why. We also know little about the type of interventions provided or their outcomes and the subsequent pathway taken by patients. Our view of key issues outside of the health sector, in areas such as housing, education and employment, is very limited. We are not able to compare or benchmark services, meaning that our system of accountability fails to impel systemic quality improvement.

As if accountability for mental health was not complex enough, past decades have seen mental health subject to multiple reforms and overlapping reporting processes. This paper traces this history and its impact on Australia’s efforts to establish effective accountability across two national mental health policies, five national mental health plans, one national action plan, several other national documents, one roadmap and multiple statutory inquiries over the past three decades. More recently, COVID-19 has seen Australia’s Federal government establish a new National Cabinet, scrapping previous administrative structures which oversaw accountability for mental health, such as the Australian Health Ministers Council [2].

Federal, state and territory governments are currently arranging bilateral agreements which will constitute the backbone of Australia’s sixth national mental health plan, including specifying data and reporting obligations. It would be folly to assume the utility of existing reporting arrangements. Indeed, under the maxim ‘what gets measured gets done’, there is reason to be alert to the risk of poor data collection processes reinforcing undesirable models of care. For example, if hospital beds are the currency reported, beds will remain the priority for policy and funding, regardless of the merits of alternatives.

Understanding Australia’s historical approach to mental health reporting can inform the next steps and help drive the development of more robust processes designed to deliver national accountability.

## 2. Materials and Methods

While no formal evaluation of Australia’s National Mental Health Strategy has occurred, this does not mean that there is a paucity of evidence. Comments and critiques are plentiful, generated by the frequent statutory, parliamentary and other inquiries commonplace over the past two decades. One report suggested that there had been thirty-two separate statutory or other inquiries between 2006 and 2012 alone [3]. Such inquiries relying on qualitative or summative evidence have often been initiated in response to deaths, human rights abuses or other tragedies. While they do not purport to formally evaluate the National Mental Health Strategy as a whole, they frequently touch on accountability and monitoring, making them worthy of consideration and review here.

In this context, this paper has relied on a narrative review, aiming to present a comprehensive, critical analysis of current knowledge in relation to Australia’s approach to reporting and accountability for mental health. It is possible, on this basis, to discern gaps and patterns, as well as strengths and weaknesses, in the data [4].

Key documents, policies, plans and strategies are considered, demonstrating the evolution of mental health data and reporting. Historical documents are cited, including several which highlight implications arising from our federated system of government. Government and statutory reports, as well as peer-reviewed and other literature (from grey literature, websites, media sources, etc.) are referenced. The jumble of reports and inquiries needs a timeline to orient readers, and this is presented. The paper explores recent recommendations made by various reports and how these can influence the direction of future reforms. It then draws on contemporary literature to describe the components of an effective, contemporary approach to accountability for mental health.

### What Is Meant by Accountability?

Accountability is an elusive concept, with multiple valid perspectives [5]. Planners would like to know the value for money. Service providers wish to understand if their work has been effective and how it could be improved. Consumers and families want to know what services and treatments work. Funders want information about cost-effectiveness and value for money, using systems such as activity-based funding to generate costs and prices and monitor system efficiency [6,7]. Researchers will want data to evaluate or compare alternative approaches, programs or services.

The community more generally will want information indicating the extent to which it has access to a mental health system that responds to individual needs and is one on which it can rely.

In relation to health care generally, accountability can increase the effectiveness of services, reduce inefficiency and provide the feedback necessary to impel systemic quality improvement [8,9].

The data generated for accountability are commonly considered across three dimensions: financial, performance and political/democratic [10]. Financial accountability relates to ensuring that funds are spent as agreed, monitoring, auditing and budgeting.

Performance accountability can refer to the assessment of services, outputs or outcomes, allowing value for money to be assessed. Political accountability is often focused on whether governments kept their promises, often with reference to notions of equity, efficiency and so on.

These perspectives on accountability overlap, but stakeholders may prioritise data differently. This diversity again lends itself to the narrative method of analysis used throughout this discussion.

## 3. Results

### 3.1. Initial Efforts in Mental Health Information

The 1992 National Mental Health Strategy, which included an overarching policy and a plan, had data and accountability at its heart (Box 1).

Box 1 **Extract from 1992 National Mental Health Policy.**

There needs to be greater accountability and visibility in reporting progress in implementing the new national approach to mental health services. Currently mental health data collection is inconsistent and would not be adequate to enable an assessment to be made of the relative stage of development of the Commonwealth and each State/Territory Government in achieving the objectives outlined in the National mental health policy. It is essential that such a consistent system of monitoring and accountability be created.

         *National Mental Health Policy (Commonwealth of Australia 1992)*

The aim of this novel approach to accountability for mental health was to report on the progress being made by governments against the Strategy’s agreed goals.

The Australian Health Ministers’ Council established a working group to oversee the implementation of the Strategy. The National Mental Health Working Group was comprised of representatives from each state and territory, plus two from the Federal government, as well as the chair and deputy chair of the newly established National Community Advisory Group, which included consumers and carers. This working group established a set of 49 indicators to fulfil the accountability monitoring function recommended in the policy.

However, the data required to report against many of these indicators either did not exist or were not collected. The working group established a Mental Health Information Strategy Sub-Committee (MHISSC) [11] comprised of the same representation as the working group plus representatives from the Australian Bureau of Statistics (ABS), the Australian Institute of Health and Welfare (AIHW) and the Australian Private Hospitals’ Association. The MHISSC developed a National Mental Health Data Dictionary and Minimum Data Set for Australia.

The MHISSC oversaw the development of a specific new data collection process designed to fulfil the Working Group’s mental health reporting obligations under the Policy. This was conducted outside the structures established already by the National Health Information Agreement, which provided the framework for establishing national data collections and data standards [12].

The Federal government engaged consultants to manage the process of collecting and analysing data, and then published a series of National Mental Health Reports [13] to draw together material from all jurisdictions, as well as the private sector.

After a baseline was established in 1993, the first report was published in 1994 [14]. By the time the Commonwealth decided to cease the series, twelve editions had been produced. The final National Mental Health Report (2013) used 18 graphs or tables to describe the pace of reform [13].

This report, produced separately from other existing health data and by external consultants, became the key tool by which the community could track changes in the shape and nature of mental health care. Drawing on the definition provided earlier, the National Mental Health Report series had a clear focus on political accountability, purporting to enable governments to answer the question “Did we do what we agreed?” [13].

Over time, the collection and report became more robust, with data elements incorporated into different national minimum datasets [15]. It reflected a strong focus on the role of the states and territories as the main providers of care, for example, in delivering the policy goal of ‘mainstreaming’ mental health services.

The reporting also had a heavy emphasis on financial accountability, as described earlier, reporting inputs such as spending and staffing, and outputs, as well as administrative data, such as treatment days, the number of services and clients.

The collection was not designed to drive a process of systemic quality improvement, nor reflect perspectives on accountability held by mental health stakeholders, such as consumers or even health professionals. Stakeholders from across the mental health sector and outside of the government would prioritise accountability issues and questions different to those selected by the government [16].

### 3.2. Limited Aims, Limited Performance

The pursuit of even this rather limited dataset was challenging enough—obtaining agreement on data collection standards and definitions between nine Australian jurisdictions is difficult. The process requires consensus across governments [17].

MHISSC then had to oversee the process by which each government obtained, vetted and cleaned the necessary data. This governmental approval was a slow process, causing delays in publication. For example, the data published in the 2013 National Mental Health Report pertained to the 2010–2011 financial year. This lag has not improved. In 2022, the AIHW’s Mental Health Services in Australia website [18], now the key data resource, is still only able to report mental health expenditure up until 2018–2019.

There was no independent verification of the data provided to the Report and, particularly in the first years, the quality and range of data varied between jurisdictions. There was no way to marry annual mental health budget allocations to the actual expenditure or to the costs of services. These matters limited the extent to which data could be usefully interpreted for benchmarking between jurisdictions.

The data were only published at the jurisdictional level (i.e., by state and territory). This could be useful, revealing how the shape and nature of the mental health services available differ between the states. For example, the 2013 National Report showed that Tasmania offered 19.5 beds per 100,000 inhabitants in residential mental health care settings, while Queensland provided zero. However, the Report had no capacity to provide data at more disaggregated levels, preventing a more detailed and regional comparison of service patterns or other issues [13].

The 1997 Evaluation of the first national mental health plan, while noting the role of the National Mental Health Report, stated:

*Information in mental health is grossly undeveloped. The lack of nationally comparable data on service outputs, costs, quality and outcomes places major limitations on the extent to which the National Mental Health Strategy can achieve its objectives.*

[19]

An initial $135 m investment made by the then Federal Government to sponsor reform and accountability under the First Plan was not replicated in subsequent plans [20].

Key proponents of the national reforms noted that, under the Second National Mental Health Plan, momentum “waned” [21].

A decade later, the ‘summative’ evaluation of the 3rd National Mental Health Plan (2003–2008) repeated concerns about national monitoring and reporting mechanisms, suggesting that there was duplication, waste and an inability to measure appropriate outcome measures [22].

These concerns about data and accountability processes in mental health were echoed in repeated statutory reports and inquiries [23,24]. A report jointly prepared by the Human Rights and Equal Opportunity Commission and the [then] Mental Health Council of Australia found:

*The National Mental Health Strategy was developed over a decade ago to respond to obvious service failures and human rights concerns….we do not yet have a national process for translating the policy rhetoric into real increases in resources, enhanced service access, accepted service standards or service accountability.*

[25]

### 3.3. Fragmentation of Effort, Minimal Improvement

The ownership of responsibility for national mental health reporting shifted in 2006 from health ministers to first ministers, with the Council of Australian Governments (CoAG) agreeing to a $5.5 bn National Action Plan on Mental Health [26]. The rationale for the CoAG’s involvement is not entirely clear. There were two damning inquiries which required some political response [23,25]. The CoAG itself reported that its engagement was based on “a broad recognition that renewed government effort was needed to give greater impetus to the reform process” [26]. The Action Plan brought together the heads of all governments to focus on mental health for the first time and included its own list of outcomes and progress measures.

The CoAG’s list had greater emphasis on social indicators, such as employment and education, than the mental health service indicators prioritised by the MHISSC. It also reflected greater engagement by the Federal government in mental health service provision. The CoAG Action Plan generated progress reports, again designed for the government to fulfil a level of political accountability and demonstrate “Are we doing what we said we would?” [27].

There were several other reports and inquiries into mental health emerging in quick succession that recommended changes to the way data are reported, or even proposed new sets of indicators [24,28] (see Table A1 for a timeline). These recommendations were not actioned.

The process of providing national accountability oversight in mental health has become increasingly confused with multiple overlapping initiatives, policies, plans and datasets. This has dramatically increased the gap between planning and reporting, and actual action and monitoring of mental health. Key processes identified as part of effective policy development and evaluation are missing [29].

The 2012 National Mental Health Roadmap, for example, listed 11 ‘performance’ indicators and 3 ‘contextual’ indicators [30]. The 4th National Mental Health Plan and associated Implementation and Measurement Strategies listed 25 indicators [31]. It continued the CoAG’s emphasis on broader measures of the social determinants of mental health, promising a “whole of government approach” so that:

The public is able to make informed judgements about the extent of mental health reform in Australia, including the progress of the fourth plan, and has confidence in the information available to make these judgements. Consumers and carers have access to information about the performance of services responsible for their care across the range of health quality domains and are able to compare these to national benchmarks [32].

The National Mental Health Commission began in 2012 and soon produced its own annual National Mental Health Report [33] drawing on frameworks, indicators, case studies and stories, rather than against a consistent dataset. In 2014, the Commission was tasked with a review of mental health programs and services and reported, in 2015, on a lack of outcome-based evaluation data and accountability mechanisms [34]. It recommended a focus on a much smaller number of indicators, focusing much more on outcomes than outputs, together with a transition to a much more regionally based system of planning and reporting. The Commission’s recommendations remain unimplemented.

The impetus towards greater accountability in mental health in relation to its social determinants was affirmed in the 2014 strategic plan of the NSW Mental Health Commission, which reported that spending on mental health by the NSW Department of Family and Community Services was greater than that by the NSW Department of Health [35]. Accountability for health care alone cannot provide a true picture of mental health.

Despite this, the 5th National Mental Health and Suicide Prevention Plan [36] and its accompanying Implementation Plan (2017) [37] promised monitoring and reporting around a more limited set of 24 core health indicators, focusing on safety and quality.

This Plan promised to draw on proxy data to deal with social determinant issues as part of this, for example, using the Australian Bureau of Statistics General Social Survey to report the social participation of people with a mental illness.

Leaving aside issues such as resources or political will, the infrastructure to support good data collection in mental health has been slow to evolve. Several other countries have developed sophisticated maps [38], permitting benchmarking and the comparison of key mental health services between jurisdictions. Such maps are new to Australia and are not yet driving decision-making. Alternative classifications and structures, such as the Australian Classification of Health Interventions (ACHI), have been demonstrated to be less than comprehensive when applied to mental health [39].

The history of Australian efforts in relation to data collection and reporting has left us with at best a partial picture—strong in relation to health and administrative data, but weak in other areas, particularly outside of hospitals and in relation to the broader social determinants of mental health. It is a situation described as “outcome blind” [40].

### 3.4. Other Key Reporting Mechanisms in Mental Health

There are two other key sources of mental health data in Australia. Unlike the National Strategy reporting, both have demonstrated some consistency.

The Australian Institute of Health and Welfare (AIHW) has published the Mental Health Services in Australia (MHSIA) data series since 1988–1999 [18], drawing on the National Mental Health Data Dictionary and Minimum Data Set originally developed by the MHISSC.

Other national minimum data sets have been developed and become part of MHSIA reporting, including in relation to:

- Mental health establishments;
- Admitted patient care;
- Residential mental health care;
- Community mental health care;
- Causes of death (for suicide data).

In 2021, this array of data permits the publication of 35 tables of information. The AIHW also hold and manage an ‘indicator library’ [41] from which they derived a set of 26 Key Performance Indicators, including issues such as rates of seclusion and restraint, rates of access to mental health care, community contact pre- and post-discharge, etc. [42]. The AIHW was also the manager of the National Mental Health Performance Framework [43] until the cessation of the CoAG in 2020.

The Productivity Commission prepares the Report on Government Services which, for 25 years, has included a section on mental health services [44,45]. Around 60 tables of information are published each year online, providing data at the state and territory levels across 13 key indicators.

There is considerable overlap across the AIHW and Productivity Commission reporting—they both provide data on public mental health service data, expenditure, staffing and access. Additionally, both publications focus on the health service aspect of mental health care, rather than the broader social determinants. They use proxy data derived from general community survey information to estimate and report on matters such as housing and employment. Both suffer from considerable delays in publication. They report progress at the jurisdictional level, permitting, for example, a comparison of the proportion of all mental health-related emergency department presentations in public hospitals between Western Australia and Tasmania. The work of the AIHW and the Productivity Commission in reporting mental health data, even at this level, is helpful, but, as recommended by the Productivity Commission Review (see below), more useful comparisons need to be established between regions, not between states [46]. This more granular approach reflects the fact that regions may have more in common and provide more valid benchmarks than comparing whole jurisdictions, such as Victoria and NSW.

### 3.5. The Productivity Commission Review 2020

The report found duplication and a lack of clarity in mental health reporting arrangements and called for all governments to agree on a new set of realistic measures and outcomes. It suggested a new framework with six key areas and 47 identified indicators [46].

This was echoed by the Victorian Royal Commission, which reported in 2021 that:

*System leadership is weak, and accountability for how the system is managed is unclear.*

[47]

These findings are obviously a strong indictment of the approach taken in Australia so far.

Under various reporting structures, the MHISSC operated continuously until the Council of Australian Governments (CoAG) was disbanded in May 2020 in favour of new National Cabinet reporting arrangements. Thus far, these arrangements seem rudimentary.

Eleven general health issues are listed under a ‘Performance Reporting Dashboard’, of which one pertains to mental health. However, rather than provide any data or indicators, what is presented is simply a list of some projects undertaken in each jurisdiction under a green tick symbol and the word “Achieved” [48].

The final National Mental Health Report was published in 2013. There have been no evaluations of either the 4th or 5th National Mental Health Plans and, as stated, no evaluation of the Strategy overall. Despite the regular calls for annual and transparent reporting and monitoring of progress, there is no current system or process for this to occur.

In 2021, the Federal Government released its response to the Productivity Commission report [49], undertaking with the states and territories to establish a new National Agreement on Mental Health and Suicide Prevention by November 2021.

## 4. Discussion

### Lessons Learned—Towards a Better Process of Accountability and Planning

From the experiences of the past thirty years, several important trends and challenges have emerged in relation to how Australia and other nations can engineer more effective and useful data collection and accountability for mental health. In recognition of the increasing role and potential of primary and community-based mental health care, new datasets continue to emerge, requiring intelligent amalgamation with existing systems to exploit new opportunities [50,51]. There is merit in considering how these issues might shape a new process or framework for mental health reporting and planning.

Improved reporting must finally accept the significance of understanding not just basic inputs and outputs, but the whole mental health ‘ecosystem’ [52], drawing on a broader set of metrics which properly reflect the mental health and wellbeing of communities. This poses new problems in organising and gathering requisite data from multiple agencies, not just health departments. The coordination of this kind of whole-of-government monitoring was one rationale for several jurisdictions to establish mental health commissions [53].

As stated, the issue of regional data is increasingly recognised as key to enabling better local planning in mental health. Despite commitments made to establish regular benchmarking in mental health over past decades [20,54], the establishment and reporting of data at this level is not yet a feature of mental health reporting in Australia, though the AIHW publication of Medicare data by statistical local area (SLA) is an exception [55]. Australia’s failure to develop a suitable mental health performance management framework with agreed, consistent indicators and targets has been pinpointed as a key drawback to reform [46].

Engaging mental health stakeholders in developing such a framework would build an understanding of the process and confidence in the results [56]. To date, MHISSC and associated governments have been largely responsible for determining how mental health is reported. MHISSC relied for twenty years on external consultants to manage the process of data collection and reporting [57]. The benefits of broadening this process have been recognised [46]. Specific mention must be made of consumers and carers in this context. The National Community Advisory Group (NCAG) mentioned earlier was disbanded after just three years in 1996. Structures to engage consumers and carers in framework co-design will require considerable development [58].

Another design element should be the widespread use of new personal technologies which permit services users to be the key reporters of real-time and local data pertaining to their care [59], as has already been demonstrated both in Australia [60] and elsewhere [61]. This should be part of a fundamental re-design of accountability for mental health, one that recognises the broader social context of mental illness beyond health, considering issues such as employment, education completion and social connectedness. Despite some initiatives [62], Australia still lacks a validated, national collection of the experience of care of mental health consumers and carers.

Finally, the way mental health is reported relates to how it is planned, and this is a matter currently up for national debate. Historic, centralised approaches to planning are being challenged by more local or regional models of governance and decision-making, as encouraged by the Productivity Commission [46] and the National Mental Health Commission.

There are new decision-support systems which enable this local planning and modelling [63,64,65]. There are clearly limitations in the extent to which existing state and territory-focused mental health data collections can provide the information these new models need to facilitate better local decision-making, or what other information might be necessary. The examination and resolution of these issues is a key element of more effective planning and reporting of mental health care.

Key bodies internationally have recognised the inability of existing mental health data systems to propel the desired processes of benchmarking and quality improvement [66]. They have embarked on projects designed to make mental health data systems more robust and useful. The World Health Organisation has, for example, prioritised the creation of a mental health data platform aiming at routinely collected information on mental health systems’ performance and on the mental health status of the population. These Australian lessons could inform this work [67].

The Australian experience demonstrates the importance of establishing an accurate historical account of the evolution of the core policy and planning processes underpinning mental health reform, giving context and meaning to the status of national and regional mental health systems. Our experience has shown how complicated this process can be, even in countries with significant resources.

## 5. Conclusions

Australia has not produced a comprehensive report or evaluation of its national mental health planning effort. This means that, despite myriad plans and reports, it is not possible to assess the extent to which this work has translated into effective change, the costs, nor the impact on individual outcomes or systemic improvement.

Even where partial data have been reported, there was no independent verification of the data provided and, particularly in the first years, the quality and range of data varied between jurisdictions. There was no way to marry annual mental health budget allocations to actual expenditure or to the costs of services. These matters limited the extent to which data could be usefully interpreted for benchmarking between jurisdictions. Australia has lacked consistent data sets. Overlapping reports, indicator sets and report cards have perpetuated confusion, not clarity.

The 1992 National Mental Health Reform Strategy had broad aspirations and called for reporting on areas of consumer and carer rights, legislation and other matters. Unable to meet the challenge of this breadth, initial reporting focused on the regular publication of mostly public mental health service activity data and related issues, such as expenditure and staffing. Some resources were provided initially to support the reporting process, but these were discontinued. This limited the further expansion of the reporting process.

As new plans emerged, the focus of mental health reforms shifted, seeking to consider issues beyond the health system. Since the CoAG in 2006, the reporting process has been subject to increasing pressure as competing policies and plans frequently emerged.

The initial clarity of purpose became confused. Commitments to better accountability were made, but resources were not provided. Mental health reporting has been managed and proceeded largely unchanged under MHISSC, leaving other mental health stakeholders outside the design process. All these factors have contributed to making the mental health data collection and reporting process less relevant over time.

Other existing mental health reporting mechanisms provided by the AIHW and the Productivity Commission focus on health services and operate without set targets. Neither impels identifiable processes of quality improvement. The new National Cabinet reporting arrangements established under COVID have diminished the mental health accountability obligations of all governments.

In 2020, the AIHW sought stakeholder views regarding a future National Health Information Strategy, considering issues such as data collection, access, reporting, privacy and so on, but without specific reference to mental health data. In response, some stakeholders suggested an urgent need for patient-reported outcome measures in mental health [68]. Mental Health Australia, the peak body, did not provide a submission to the AIHW. The separation of mental health data development from the rest of health has been a defining feature of the past thirty years of Australia’s mental health strategy. There are clearly risks that this unhelpful separation could continue.

As of early 2022, the Australian government has been announcing a series of bilateral agreements with each of the eight states and territories which will form the backbone of the sixth National Mental Health and Suicide Prevention Plan [69]. Details of these arrangements, including data collection and reporting obligations, are yet to be made public.

The establishment of an entirely new accountability framework was a key recommendation of the Productivity Commission [46]. This framework will need to facilitate new approaches to regional modelling, governance and reporting across the whole ‘ecosystem’ of mental health. It will require expertise and resources. It must be based on a robust process of co-design, properly accounting for the different, but related, needs of planners, funders, service providers, consumers, carers, researchers and others. These are the ingredients for effective systemic oversight and local quality improvement. Such a system can build new community trust in our mental health system.

## Appendix A

**Table A1:** Mental health data and accountability timeline.

Year	Policy Document	Notes in Relation to Data/Accountability
1992	First National Mental Health Strategy (and Policy)	
1993–1998	First National Mental Health Plan	Eight areas identified
1994	First National Mental Health Report	Established baseline
1995–2013	National Mental Health Report Series—11 editions	In 2013, 24 national indicators plus 18 indicators reported at jurisdictional level
1995	First Report on Government Services (ROGS) by Productivity Commission	2021 edition includes 60 tables of information (most recent year reported is 2018–2019).
1997	Evaluation of the First National Mental Health Plan	
1998-2003	Second National Mental Health Plan	
2001	First Mental Health Services in Australia report published by the Australian Institute of Health and Welfare.	2021 edition includes 35 tables of information (most recent year reported is 2018–2019).
2001	International Mid-Term Review of the Second National Mental Health Plan	
2003	Evaluation of the Second National Mental Health Plan	
2003–2008	Third National Mental Health Plan	34 outcomes, 113 key directions.
2005	National Mental Health Report (9th)	Summary of 10 Years of the National Mental Health Reform Strategy
2005	First National Mental Health Performance Framework	
2006–2011	Council of Australian Governments’ National Action Plan on Mental Health	12 progress measures
2008	Evaluation of the Third National Mental Health Plan	
2009	Second National Mental Health Policy	Replacing the original 1992 document.
2009–2014	Fourth National Mental Health Plan	
2009	National Advisory Council on Mental Health	Recommended changing accountability framework for mental health (not actioned).
2010	Fourth National Mental Health Plan Implementation Strategy	
2011	Fourth National Mental Health Plan Measurement Strategy	5 key areas, 27 indicators.
2012–2022	Council of Australian Governments’ National Roadmap for Mental Health Reform	11 ‘performance’ indicators and 3 ‘contextual’ indicators.
2012	National Mental Health First Report Card—A Contributing Life	Seven key areas reported.
2014	National Mental Health Commission Review—Contributing Lives, Thriving Communities	Eight key indicators/targets identified for new reporting framework (not actioned).
2015	Australian Government Response to National Commission Review	Undertaken to develop new indicators as part of 5th National Mental Health Plan
2017	5th National Mental Health and Suicide Prevention Plan	24 indicators focusing on quality and safety
2020	Productivity Commission Report into Mental Health	6 key areas with 47 indicators recommended
2020	National mental health and wellbeing pandemic response plan	[Committed] to data collection and modelling, and the development of indicators for informed policy development
2020	CoAG process disbanded in favour of National Cabinet	
2021	Victorian Royal Commission into mental health	Recommended establishment of new regional mental health indicators under a Mental Health and Wellbeing Outcomes Framework
2021	Prevention, Compassion, Care—National Mental Health and Suicide Prevention Plan	Committed to developing new a National Agreement on Mental Health and Suicide Prevention between all governments.
